# Author Correction: Inference and reconstruction of the heimdallarchaeial ancestry of eukaryotes

**DOI:** 10.1038/s41586-026-10115-4

**Published:** 2026-02-11

**Authors:** Laura Eme, Daniel Tamarit, Eva F. Caceres, Courtney W. Stairs, Valerie De Anda, Max E. Schön, Kiley W. Seitz, Nina Dombrowski, William H. Lewis, Felix Homa, Jimmy H. Saw, Jonathan Lombard, Takuro Nunoura, Wen-Jun Li, Zheng-Shuang Hua, Lin-Xing Chen, Jillian F. Banfield, Emily St John, Anna-Louise Reysenbach, Matthew B. Stott, Andreas Schramm, Kasper U. Kjeldsen, Andreas P. Teske, Brett J. Baker, Thijs J. G. Ettema

**Affiliations:** 1 Department of Cell and Molecular Biology, Science for Life Laboratory, Uppsala University, Uppsala, Sweden; 2 Laboratoire Écologie, Systématique, Évolution, CNRS, Université Paris-Saclay, AgroParisTech, Gif-sur-Yvette, France; 3 Laboratory of Microbiology, Wageningen University and Research, Wageningen, The Netherlands; 4 Department of Aquatic Sciences and Assessment, Swedish University of Agricultural Sciences, Uppsala, Sweden; 5 Department of Marine Science, Marine Science Institute, University of Texas Austin, Port Aransas, TX USA; 6 Research Center for Bioscience and Nanoscience (CeBN), Japan Agency for Marine-Earth Science and Technology (JAMSTEC), Yokosuka, Japan; 7 State Key Laboratory of Biocontrol, Guangdong Provincial Key Laboratory of Plant Resources and Southern Marine Science and Engineering Guangdong Laboratory (Zhuhai), School of Life Sciences, Sun Yat-Sen University, Guangzhou, PR China; 8 Chinese Academy of Sciences Key Laboratory of Urban Pollutant Conversion, Department of Environmental Science and Engineering, University of Science and Technology of China, Hefei, PR China; 9 Department of Earth and Planetary Sciences, University of California, Berkeley, CA USA; 10 Department of Environmental Science, Policy, and Management, University of California, Berkeley, CA USA; 11 Department of Biology, Portland State University, Portland, OR USA; 12 School of Biological Sciences, University of Canterbury, Christchurch, New Zealand; 13 Section for Microbiology, Department of Biology, Aarhus University, Aarhus, Denmark; 14 Department of Earth, Marine and Environmental Sciences, University of North Carolina, Chapel Hill, NC USA; 15 Present Address: Theoretical Biology and Bioinformatics, Department of Biology, Faculty of Science, Utrecht University, Utrecht, The Netherlands; 16 Present Address: Department of Biology, Lund University, Lund, Sweden; 17 Present Address: Department of Integrative Biology, University of Texas Austin, Austin, TX USA; 18 Present Address: Structural and Computational Biology, European Molecular Biology Laboratory, Heidelberg, Germany; 19 Present Address: Department of Marine Microbiology and Biogeochemistry, NIOZ, Royal Netherlands Institute for Sea Research, AB Den Burg, The Netherlands; 20 Present Address: Department of Biochemistry, University of Cambridge, Cambridge, UK; 21 Present Address: Department of Biological Sciences, The George Washington University, Washington, DC USA

**Keywords:** Phylogenetics, Archaeal evolution, Metagenomics

Correction to: *Nature* 10.1038/s41586-023-06186-2 Published online 14 June 2023

Our manuscript included a phylogenomic study of the evolutionary relationship between eukaryotes and Asgard archaea, showing that eukaryotes likely emerged from a bona fide Asgard archaeal ancestor. Our results suggested that eukaryotes and the heimdallarchaeial order Hodarchaeales form a monophyletic group. A set of 57 phylogenetic markers (NM57) was central to reach these conclusions. After the publication of our study, we noticed that three of these markers were partially redundant, as they belong to paralogous families. We have therefore reduced this dataset to 54 non-redundant markers (NM54; we removed markers M127, M028, and MA54) and used the same methodology to re-run all phylogenomic analyses presented in the paper.

The results of the analyses of the corrected dataset are consistent with the original findings: while we observe small variations in statistical support, the overall trends support that eukaryotes are placed within Heimdallarchaeia, as sister-group to the order Hodarchaeales when both Susko-Roger-4 (SR4)-recoding and Fast-site removal (FSR) treatments were combined.

A full discussion of changes to the article is available as Supplementary information accompanying this amendment. Text, figures and Supplementary information have been amended in the HTML and PDF versions of the article.

**Supplementary information** is available in the online version of this amendment.

## Supplementary information

Discussion of changes to the original article

Annotated changes to original article and uncorrected Supplementary Figs.

