# Supplementary material for: Inference and reconstruction of the heimdallarchaeial ancestry of eukaryotes
Source: Nature. 2023 Jun 14;618(7967):992–9. doi: 10.1038/s41586-023-06186-2 (PMC10307638; doi:10.1038/s41586-023-06186-2)
Supplement: Supplementary file 2 — Reporting Summary [file 41586_2023_6186_MOESM2_ESM.pdf]

nature research

Corresponding author(s): Thijs. J. G. Ettema

Last updated by author(s): Apr 20, 2023

Reporting Summary

Nature Research wishes to improve the reproducibility of the work that we publish. This form provides structure for consistency and transparency in reporting. For further information on Nature Research policies, see our Editorial Policies and the Editorial Policy Checklist.

Statistics

For all statistical analyses, confirm that the following items are present in the figure legend, table legend, main text, or Methods section.

n/a	Confirmed	
☐	☒	The exact sample size (*n*) for each experimental group/condition, given as a discrete number and unit of measurement
☒	☐	A statement on whether measurements were taken from distinct samples or whether the same sample was measured repeatedly
☐	☒	The statistical test(s) used AND whether they are one- or two-sided<br>*Only common tests should be described solely by name; describe more complex techniques in the Methods section.*
☒	☐	A description of all covariates tested
☒	☐	A description of any assumptions or corrections, such as tests of normality and adjustment for multiple comparisons
☐	☒	A full description of the statistical parameters including central tendency (e.g. means) or other basic estimates (e.g. regression coefficient) AND variation (e.g. standard deviation) or associated estimates of uncertainty (e.g. confidence intervals)
☐	☒	For null hypothesis testing, the test statistic (e.g. *F*, *t*, *r*) with confidence intervals, effect sizes, degrees of freedom and *P* value noted<br>*Give P values as exact values whenever suitable.*
☐	☒	For Bayesian analysis, information on the choice of priors and Markov chain Monte Carlo settings
☒	☐	For hierarchical and complex designs, identification of the appropriate level for tests and full reporting of outcomes
☒	☐	Estimates of effect sizes (e.g. Cohen's *d*, Pearson's *r*), indicating how they were calculated

Our web collection on statistics for biologists contains articles on many of the points above.

Software and code

Policy information about availability of computer code

Data collection	No software was used for data collection.
Data analysis	Custom scripts have been deposited on Github (https://github.com/laurajjeme/phylogenetics). Published software used for data analysis include BBTools v38.79, Sickle v1.33, metaSPAdes v3.10.1, MetaBAT v2.12.1, Trimmomatic v.0.36, MEGAHIT v.1.1.1-2-g02102e1, SeqTK v1.0r75, CONCOCT v0.4.1, CLARK v1.2.3, miComplete v1, mmgenome v0.7.1, IDBA-UD 1.1.3, cutadapt v1.12, CheckM v1.0.5, IQ-TREE v. 2.0-rc2, Prokka v1.12, SiLiX v.1.2.10, Hifix v1.0.6, HHblits v3.0.3, Interproscan 5.25-64.0, EggNOG mapper v0.12.7, GhostKoala,

For manuscripts utilizing custom algorithms or software that are central to the research but not yet described in published literature, software must be made available to editors and reviewers. We strongly encourage code deposition in a community repository (e.g. GitHub). See the Nature Research guidelines for submitting code & software for further information.

Data

Policy information about availability of data

All manuscripts must include a data availability statement. This statement should provide the following information, where applicable:

- Accession codes, unique identifiers, or web links for publicly available datasets
- A list of figures that have associated raw data
- A description of any restrictions on data availability

The MAGs reported in this study have been deposited at DDBJ/EMBL/GenBank. BioProject IDs, BioSample IDs and GenBank assembly accession numbers are available in Supplementary Table 1. All raw data underlying phylogenomic analyses (raw and processed alignments and corresponding phylogenetic trees), and all predicted proteomes have been deposited on Figshare (10.6084/m9.figshare.22678789).

Field-specific reporting

Please select the one below that is the best fit for your research. If you are not sure, read the appropriate sections before making your selection.

☒ Life sciences ☐ Behavioural & social sciences ☐ Ecological, evolutionary & environmental sciences

For a reference copy of the document with all sections, see nature.com/documents/nr-reporting-summary-flat.pdf

Life sciences study design

All studies must disclose on these points even when the disclosure is negative.

Sample size	Sample sizes (i.e. the number of lineages included in phylogenomic analyses) were empirically determined based on the computational resources necessary to run the various analyses.
Data exclusions	No data was excluded
Replication	Robustness and reliability of phylogenetic analyses were assessed using 100 bootstrap replicates for all maximum likelihood analyses, as is commonly done in the field.
Randomization	Randomization is not necessary to a study using phylogenetic approaches because these approaches rely on the comparison of evolutionary relationships between species, rather than on random assignment of treatments or control groups. Phylogenetic analyses are not affected by the same sources of bias as experimental designs, such as confounding variables or selection bias. Therefore, while randomization is a useful tool in many types of research, it is not essential in studies that using phylogenetics and comparative genomics.
Blinding	Blinding is not necessary to a study using phylogenetic approaches because these methods are based on objective comparisons of evolutionary relationships between species, rather than on subjective assessments or measurements of treatment effects. These analyses do not involve human subjects, interventions, or subjective judgments that could be influenced by knowledge of the study conditions or treatments. Therefore, blinding is not relevant to the validity or reliability of phylogenetic studies, and its use is not required or expected in this type of research.

Reporting for specific materials, systems and methods

We require information from authors about some types of materials, experimental systems and methods used in many studies. Here, indicate whether each material, system or method listed is relevant to your study. If you are not sure if a list item applies to your research, read the appropriate section before selecting a response.

Materials & experimental systems

n/a	Involved in the study	
☒	☐	Antibodies
☒	☐	Eukaryotic cell lines
☒	☐	Palaeontology and archaeology
☒	☐	Animals and other organisms
☒	☐	Human research participants
☒	☐	Clinical data
☒	☐	Dual use research of concern

Methods

n/a	Involved in the study	
☒	☐	ChIP-seq
☒	☐	Flow cytometry
☒	☐	MRI-based neuroimaging
