# Supplementary material for: Author Correction: Inference and reconstruction of the heimdallarchaeial ancestry of eukaryotes
Source: Nature. 2026 Feb 11;651(8104):E1. doi: 10.1038/s41586-026-10115-4 (PMC12960242; doi:10.1038/s41586-026-10115-4)
Supplement: Supplementary file 1 — Discussion of changes to the original article [file 41586_2026_10115_MOESM1_ESM.pdf]

Supplementary Information to Author Correction: Inference and reconstruction of the heimdallarchaeial ancestry of eukaryotes

Laura Eme[1,2]▲, Daniel Tamarit[1,3,4]▲†, Eva F. Caceres[1,3]▲, Courtney W. Stairs[1]††, Valerie De Anda[5], Max E. Schön[1], Kiley W. Seitz[5]†††, Nina Dombrowski[5]††††, William H. Lewis[1,3]†††††, Felix Homa[3], Jimmy H. Saw[1]††††††, Jonathan Lombard[1], Takuro Nunoura[6], Wen-Jun Li[7], Zheng-Shuang Hua[8], Lin-Xing Chen[9], Jillian F. Banfield[9,10], Emily St John[11], Anna-Louise Reysenbach[11], Matthew B. Stott[12], Andreas Schramm[13], Kasper U. Kjeldsen[13], Andreas P. Teske[14], Brett J. Baker[5], Thijs J. G. Ettema[1,3*]

[1]Department of Cell and Molecular Biology, Science for Life Laboratory, Uppsala University, 75123 Uppsala, Sweden

[2]Laboratoire Écologie, Systématique, Évolution, CNRS, Université Paris-Saclay, AgroParisTech, 91190 Gif-sur-Yvette, France

[3]Laboratory of Microbiology, Wageningen University and Research, 6708 WE Wageningen, The Netherlands

[4]Department of Aquatic Sciences and Assessment, Swedish University of Agricultural Sciences, SE-75007 Uppsala, Sweden

[5]Department of Marine Science, Marine Science Institute, University of Texas Austin, Port Aransas, TX, 78373, USA

[6]Research Center for Bioscience and Nanoscience (CeBN), Japan Agency for Marine-Earth Science and Technology (JAMSTEC), 2-15 Natsushima-cho, Yokosuka, 237-0061, Japan

[7]State Key Laboratory of Biocontrol, Guangdong Provincial Key Laboratory of Plant Resources and Southern Marine Science and Engineering Guangdong Laboratory (Zhuhai), School of Life Sciences, Sun Yat-Sen University, Guangzhou 510275, PR China

[8]Chinese Academy of Sciences Key Laboratory of Urban Pollutant Conversion, Department of Environmental Science and Engineering, University of Science and Technology of China, Hefei, 230026, PR China

[9]Department of Earth and Planetary Sciences, University of California, Berkeley, California, USA

[10]Department of Environmental Science, Policy, and Management, University of California, Berkeley, California, USA

[11]Department of Biology, Portland State University, Portland, Oregon, USA

[12]School of Biological Sciences, University of Canterbury, Christchurch, 8142, New Zealand

[13]Section for Microbiology, Department of Biology, Aarhus University, 8000 Aarhus, Denmark

[14]Department of Earth, Marine and Environmental Sciences, University of North Carolina, Chapel Hill, USA

*Correspondence to: thijs.ettema@wur.nl

▲Equal contribution

† Current address: Theoretical Biology and Bioinformatics, Department of Biology, Faculty of Science, Utrecht University, Padualaan 8, 3584CH Utrecht, The Netherlands

†† Current address: Department of Biology, Lund University, Sölvegatan 35, 223 62 Lund, Sweden

††† Current address: Structural and Computational Biology, European Molecular Biology Laboratory, Meyerhofstraße 1, 69117 Heidelberg, Germany

†††† Current address: NIOZ, Royal Netherlands Institute for Sea Research, Department of Marine Microbiology and Biogeochemistry; AB Den Burg, The Netherlands.

††††† Current address: Department of Biochemistry, University of Cambridge, Cambridge, CB2 1QW, UK

††††††: Current address: Department of Biological Sciences, The George Washington University, Washington, DC, USA

Correction to: *Nature* https://doi.org/10.1038/s41586-023-06186-2. Published online 14 June 2023.

Our manuscript included a phylogenomic study of the evolutionary relationship between eukaryotes and Asgard archaea, showing that eukaryotes likely emerged from a *bona fide* Asgard archaeal ancestor. Our results suggested that eukaryotes and the heimdallarchaeial order Hodarchaeales form a monophyletic group. A set of 57 phylogenetic markers (NM57) was central to reach these conclusions. After the publication of our study, we noticed that three of these markers were partially redundant, as they belong to paralogous families. We have therefore reduced this dataset to 54 non-redundant markers (NM54; we removed markers M127, M028, and MA54) and used the same methodology to re-run all phylogenomic analyses presented in the paper.

The results of these updated analyses are consistent with our original findings: while we observe small variations in statistical support, the overall trends remain identical, based on data investigation such as Susko-Roger-4 (SR4) recoding and/or Fast-site removal.

Accordingly, updates are included in Methods section, dataset nomenclature, Supplementary Table 2 (to reflect the re-computed support values), and Figure 2 and the Supplementary Information (showing re-computed phylogenies).

In the main text, the second, third, fourth and fifth sentence under the subheading “Eukarya emerged within Heimdallarchaeia” were updated from “However, ML analyses of the SR4-recoded datasets retrieved a complex phylogenetic signal. In some cases, eukaryotes were placed at the base of all Heimdallarchaeia (including Njordarchaeales) and Wukongarchaeia. This result strongly suggested that the previously observed phylogenetic affiliation between Njordarchaeales and eukaryotes could represent an artefact. Furthermore, when both SR4-recoding and FSR treatments were combined, eukaryotes were nested within Heimdallarchaeia as a sister group to the order Hodarchaeales (Fig. 2 and Supplementary Fig. 8).” to “However, ML analyses of the SR4-recoded datasets showed very weak statistical support for this position, strongly suggesting that the previously observed phylogenetic affiliation between Njordarchaeales and eukaryotes could represent an artefact. Furthermore, when both SR4-recoding and FSR treatments were combined, eukaryotes were nested within Heimdallarchaeia, as sister-group to the order Hodarchaeales (Figure 2; Supplementary Figure 8).”

Figure 2 was updated accordingly. The support for the phylogenetic affiliation between eukaryotes and Hodarchaeales in panel a is identical to the previously published version in Eme et al (2023), and panel b now shows the main topologies recovered for the untreated datasets, Fast-Site Removal and/or SR4 recoding. The latter is in agreement with the previous version of the figure, although we previously did not map the results for the FSR treatment only.

We have also re-run all analyses related to amino acid composition of the corrected dataset (Supplementary Information, Supplementary Figures 4–6). While, as expected, statistical support values for individual analyses varied, the overall results reflect those based on the NM57 dataset. We have updated all affected Figures and Tables in the Supplementary Information accordingly (see details below).

We have updated Figure 2, Supplementary Figures 3-6, 8, 9, 17, 18, 21, 23, 25– 27, and Supplementary Tables 1 and 2, to reflect the results of the analyses of the corrected NM54 marker set.

We also made a minor correction to Figure 3 and Supplementary Table 3 to reflect the presence of Retromer subunit Vps26 (Retromer:Vps26) in Asgardarchaeia.

Finally, we corrected some minor typos (e.g., the A64 dataset was once erroneously referred to as A68; Figure 3 was wrongly referenced as Figure 2 in the Methods section, and a few figures were wrongly referenced in the Supplementary Information file).

Changes to the article are highlighted as comments in the accompanying “Supplementary Information: Annotated changes to original article and uncorrected Supplementary Figs.” pdf file.

None of the other analyses are affected.

The text of the Supplementary Information remains identical excepted minor changes in the following sections:

- **1.1.2. The inclusion of Korarchaeota ribosomal protein sequences generates strong topological effects:** l. 209 now reads "In addition, the position of eukaryotes either remained stable after the removal of Korarchaeota (NM54-A175-nDK), as they clustered with Njordarchaeales (BS = 98%), or moved to a more nested position, as sister to Hodarchaeales (NM54-A64-nDK, BS = 75%) (Supplementary Figure 21)." instead of "In addition, the position of eukaryotes remained stable after the removal of Korarchaeota (NM57-A64-nDK and NM57-A175-nDK), as they clustered with Njordarchaeales (BS = 82% and BS = 98% for NM57-A64-nDK and NM57-A175-nDK, respectively) (Supplementary Figure 21)."
- **1.1.3. Compositional adaptations to thermostability underlie phylogenetic conflict:**
    - l. 235 now reads "For example, in the phylogeny obtained from the concatenated alignment NM54-A175-nDK, the stem branch of Njordarchaeales was 2.90 and 2.59 times longer than those of Jordarchaeia and Baldrarchaeia" instead of "For example, in the phylogeny obtained from the concatenated alignment NM57-A175-nDK, the stem branch of Njordarchaeales was 1.90 and 1.70 times longer than those of Jordarchaeia and Baldrarchaeia"
    - Metrics were updated l. 274-284. It now reads "We also observed much higher ratio values for all four lineages in the RP56 proteins (mean±standard deviation: 1.94±1.05, 2.04±1.05, 2.58±1.25 and 2.93±1.80 for Hodarchaeales, Gerdarchaeales+Kariarchaeceae+Heimdallarchaeceae, Njordarchaeales, and Korarchaeota, respectively) than in the NM54 proteins (1.32±0.48, 1.43±0.40, 1.83±1.22 and 1.92±0.87, respectively)." instead of "We also observed much higher ratio values for all four lineages in the RP56 proteins (mean±standard deviation: 1.94±1.05, 2.04±1.05, 2.58±1.25 and 2.93±1.80 for Hodarchaeales, Gerdarchaeales+Kariarchaeceae+Heimdallarchaeceae, Njordarchaeales, and Korarchaeota, respectively) than in the NM57 proteins (1.36±0.40, 1.46±0.47, 2.04±1.21 and 2.04±0.86, respectively)."
    - The statistics were updated l. 329-352, which now reads "The ratio of charged versus polar amino acids in Njordarchaeales genomes was higher, both in the RP56 and NM54 gene markers, at sites favouring the monophyly of Njordarchaeales and Korarchaeota compared to sites that favoured the monophyly of Njordarchaeales and other Heimdallarchaeia (t-test p-values of 1.49e-8 and 1.28e-5 for NM54 and RP56 gene datasets, respectively). Moreover, this ratio was notably higher in the RP56 dataset (median for sites that supported

Njordarchaeales+Korarchaeota=2.37; median for sites that supported Njordarchaeales+Heimdallarchaeia=2.09) than in the NM54 dataset (2.16 and 1.76, respectively). Similarly, the fraction ILVWYGERKP also showed significantly higher values in sites favouring the monophyly of Njordarchaeales and Korarchaeota (t-test p-values of 6.2e-12 and 7.78e-15 for NM54 and RP56 datasets, respectively), and consistently showed higher values in the RP56 dataset (median for sites that supported Njordarchaeales+Korarchaeota=0.71; median for sites that supported Njordarchaeales+Heimdallarchaeia=0.66) compared to the NM54 dataset (0.68 and 0.64, respectively)." instead of "The ratio of charged versus polar amino acids in Njordarchaeales genomes was higher, both in the RP56 and NM57 gene markers, at sites favouring the monophyly of Njordarchaeales and Korarchaeota compared to sites that favoured the monophyly of Njordarchaeales and other Heimdallarchaeia (t-test p-values of 0.0011 and 1.28e-5 for NM57 and RP56 gene datasets, respectively). Moreover, this ratio was notably higher in the RP56 dataset (median for sites that supported Njordarchaeales+Korarchaeota=2.37; median for sites that supported Njordarchaeales+Heimdallarchaeia=2.09) than in the NM57 dataset (1.86 and 1.75, respectively). Similarly, the fraction ILVWYGERKP also showed significantly higher values in sites favouring the monophyly of Njordarchaeales and Korarchaeota (t-test p-values of 2.5e-12 and 7.78e-15 for NM57 and RP56 datasets, respectively), and consistently showed higher values in the RP56 dataset (median for sites that supported Njordarchaeales+Korarchaeota=0.71; median for sites that supported Njordarchaeales+Heimdallarchaeia=0.66) compared to the NM57 dataset (0.66 and 0.63, respectively)."

In summary, the analyses of the corrected, non-redundant NM54 dataset corroborate that the finding of the emergence of eukaryotes within Heimdallarchaeia is robust, and that the NM54 dataset represents a more streamlined marker set for future phylogenomic studies, e.g., those aiming to resolve the position of eukaryotes in the tree of life. We provide updated sequence files, alignments and trees, as well as Table S2, in a new Figshare repository (https://doi.org/10.6084/m9.figshare.29436380).
